# Lesions of the Epiglottic Cartilage

**Published:** 1857-11

**Authors:** Horace Green


*Lesions of the Epiglottic Cartilage.* By HORACE GREEN, M. D., LL.D., etc.

We lately read with pleasure, the paper which is now before us, in the American Medical Monthly for October. The pathology and treatment of the diseases affecting the epiglottis, with some interesting physiological deductions, are here considered. The principal lesions are:

1st. Erosions or abrasons of its mucous surface.

2d. Ulceration of the membrane and of its glands.

3d. Œdema or infiltration of its areolar tissue.

These lesions, their diagnosis and treatment, are treated of at length. The means of diagnosis are extremely simple, being merely to bring into view the epiglottis, by forcibly depressing the tongue, when we can easily detect the nature of the difficulty if it be seated in this part. The Dr. then applies his favorite arg. nit. and every thing goes well. The cases related have all been accompanied by a teasing continuous cough, which has been referred to the lungs, larynx, or trachea, but which inspection shows to be dependent on a little insignificant abrason or ulceration of the mucous membrane covering the epiglottis, which is speedily remedied by a few applications of nitrate of silver in stick or solution. Dr. Green, among other cases, gives one which was treated by the celebrated Hahnemann; after a long trial of the infinitisimal doses without effect, though the patient was assured of success, Hahnemann advised his patient to contract syphilis and wait for the secondary effects, which he said would eradicate his present disorder! This is curing one disease by substituting another, with a vengeance, and if the patient were compelled to cure his syphilis by producing the mercurial disease, he would wish himself in good old fashioned hands from the beginning. The patient came under Dr. Green's care, who cured him by topical applications.

Several cases are related of persons who have lost the greater part, or the whole of the epiglottis without experiencing any serious difficulty in swallowing, or in speaking, showing that the epiglottis is not so essential to the protection of the windpipe, as was formerly supposed. This has long since been demonstrated in this country as well as in Europe, by excising the epiglottis of dogs; but it is seldom that we see cases in the human subject illustrative of this fact.

Dr. Green in one case saw distinctly the movements of the glottis, which sufficiently demonstrates to any one the possibility of passing an instrument through the chink, which is still denied by some. We have never seen this in the human subject, but if it be anything like the movements in the dog, there can be no difficulty in introducing a large sized instrument if we seize our opportunity. We have no kind of doubt that it is done every day and can be done by any one.

Very little is said in text-books and in the lecture room, about lesions of the epiglottic cartilage, and the deficiency has been ably supplied by Dr. Green.

